# Simultaneous Subconjunctival Triamcinolone and Bevacizumab Injections for Management of Blepharokeratoconjunctivitis in Children

**DOI:** 10.1155/2018/2602487

**Published:** 2018-06-05

**Authors:** Chu Luan Nguyen, Tony S. Chen, Khoi Tran, James E. H. Smith, Noni Lewis

**Affiliations:** ^1^Department of Ophthalmology, Royal North Shore Hospital, Sydney, NSW, Australia; ^2^Sydney Medical School, University of Sydney, Sydney, NSW, Australia

## Abstract

**Purpose:**

To report the efficacy of subconjunctival triamcinolone (Kenalog A-40, Alcon) and bevacizumab (Avastin, Genentech) injections in fraternal twins with blepharokeratoconjunctivitis (BKC) causing progressive, bilateral corneal neovascularization and scarring.

**Methods:**

In this retrospective observational case series, two three-year-old male twins with BKC had presented with bilateral red eyes, photophobia, and frequent blinking. Examination of each child showed bilateral deep stromal and superficial corneal neovascularization, corneal infiltrates, multiple follicles on the palpebral conjunctiva bilaterally with blepharitis, and thick turbid sebum expressed from the Meibomian glands. Their disease progressed despite conventional treatment. Both twins were managed with subconjunctival triamcinolone injection and subconjunctival bevacizumab injection of each eye.

**Results:**

The treatment resulted in improvement of symptoms, and examination over an 8-10-month period postinjections showed fading stromal corneal infiltrates, partially regressed corneal neovascularization, and reduced conjunctival injection without complications.

**Conclusion:**

This case series highlights the potential vision threatening complications of BKC. In addition to conventional management options, this report is the first published use of subconjunctival triamcinolone and bevacizumab injections for BKC in children in an attempt to minimize and improve corneal neovascularization and scarring and subsequently to retain useful vision.

## 1. Introduction

Blepharokeratoconjunctivitis (BKC) is a chronic, inflammatory eyelid margin disease with conjunctival and corneal involvement. Conventional treatments include lid hygiene, topical lubricants, topical and oral antibiotics, and topical corticosteroids. BKC can cause progressive corneal neovascularization and scarring despite conventional management [1]. We report the efficacy of subconjunctival triamcinolone acetonide (Kenalog A-40, Alcon) and bevacizumab (Avastin, Genentech) injections in fraternal twins with BKC.

## 2. Case Presentation

### 2.1. Case 1

Twin 1, a three-year-old Caucasian boy, was initially referred by his family physician with photophobia and frequent blinking of the left eye to Royal North Shore Hospital. He had varicella-zoster virus (VZV) keratitis of the left eye with positive VZV on Polymerase Chain Reaction (PCR) and was treated with intravenous acyclovir and topical acyclovir. His symptoms and follow-up examinations showed resolution.

**Figure 1:**
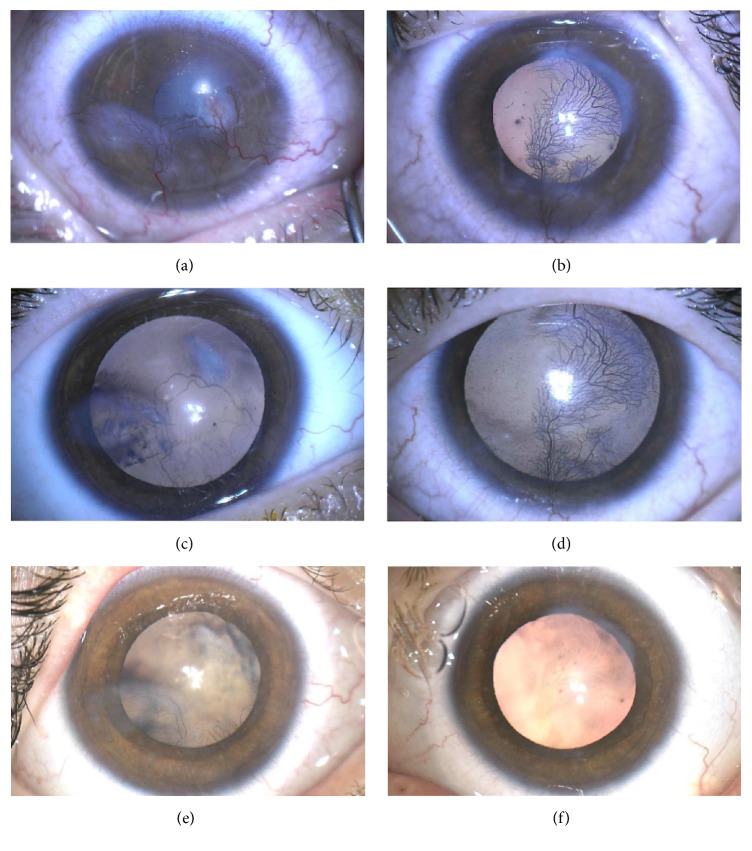
Photographs of Twin 1 with blepharokeratoconjunctivitis before and after treatment over a 10-month period. (a) Right cornea with infiltrate and overlying epithelial defect and corneal neovascularization. (b) Left cornea with central scar with active feeder vessels. (c) Right cornea eight weeks after subconjunctival triamcinolone and bevacizumab injections showing fading stromal infiltrates and regressed bloods vessels. (d) Left cornea in eight-week time with no injections showing persisting corneal neovascularization, and no observable benefit from systemic effect of bevacizumab. (e) Right cornea 10 months after bilateral subconjunctival triamcinolone and bevacizumab injections and 5 months after additional triamcinolone injections, with central corneal scarring and suppressed blood vessels. (f) Left cornea 8 months after bilateral subconjunctival triamcinolone and bevacizumab injections and 5 months after additional triamcinolone injections and diathermy with central corneal scarring and suppressed blood vessels.

One year from this presentation, best-corrected visual acuity (BCVA) in the right was 20/80 and could not be obtained in the left due to objection to occlusion. Examination under anaesthesia (EUA) revealed corneal neovascularization bilaterally (Figures 1(a)-1(b)). There was blepharitis and conjunctivitis bilaterally, with a stromal infiltrate and overlying epithelial defect in the right eye. He was investigated for causes of interstitial keratitis and corneal neovascularization. Blood tests, and corneal and conjunctival swabs were unremarkable (Appendix 1). Given the results and EUA findings the diagnosis was staphylococcal hypersensitivity/chronic blepharokeratoconjunctivitis (BKC) secondary to meibomianitis, with corneal neovascularization and scarring [2, 3]. He was prescribed oral erythromycin (160mg, twice a day), chloramphenicol ointment (1%, three times a day) for both eyes, and topical fluorometholone (0.1%, twice a day) for the left eye.

Review two weeks later showed progressive corneal neovascularization and infiltrate. Due to the progression despite conventional treatment and to aid compliance with the treatment, the decision was made for subconjunctival triamcinolone acetonide and bevacizumab, with regular EUA for treatment and monitoring of progress and side effects. Two weeks later, he received subconjunctival triamcinolone (4mg/0.1mL × 0.55mL) to the inferior conjunctiva and subconjunctival bevacizumab (2.5mg/0.1mL × 0.05mL) to the superior conjunctiva of the right eye. Extensive lid hygiene including 5% povidone-iodine lid scrubs and Meibomian gland expression was performed, and this was repeated at subsequent EUA. Intraocular pressures (IOP) by iCare® tonometer (iCare Finland) were 14mmHg in each eye. He continued on the same medication regimen and SteriLid (TheraTears®) two to three times per week.

Six weeks after the injections, the mother of Twin 1 reported significant improvement in his symptoms. At EUA eight weeks after the injections, the right cornea looked markedly improved (Figures 1(c)-1(d)). IOPs were 12mmHg in the right and 6mmHg in the left eye. Given that there had been dramatic improvement with no significant side effects observed, he was continued on the same regimen and the left eye was injected with the same dose of triamcinolone and bevacizumab.

At EUA 11 weeks after the left eye injections, there was quiescent interstitial keratitis. Adjunctive diathermy was applied to the patent superior and inferior feeder vessels in the left eye. Further subconjunctival triamcinolone (4mg/0.1mL × 0.5mL) was injected inferiorly in each eye. Binocular BCVA (due to objection to occlusion) was 20/63.

At EUA 5 months after the further bilateral triamcinolone injections, there was central corneal scarring and suppressed blood vessels bilaterally (Figures 1(e)-1(f)). Diathermy was applied at the limbus to further feeder vessels of each eye. IOP was 16mmHg in the right and 19mmHg in the left eye. Binocular BCVA (due to objection to occlusion) 24 months after presentation was 20/63.

### 2.2. Case 2

**Figure 2:**
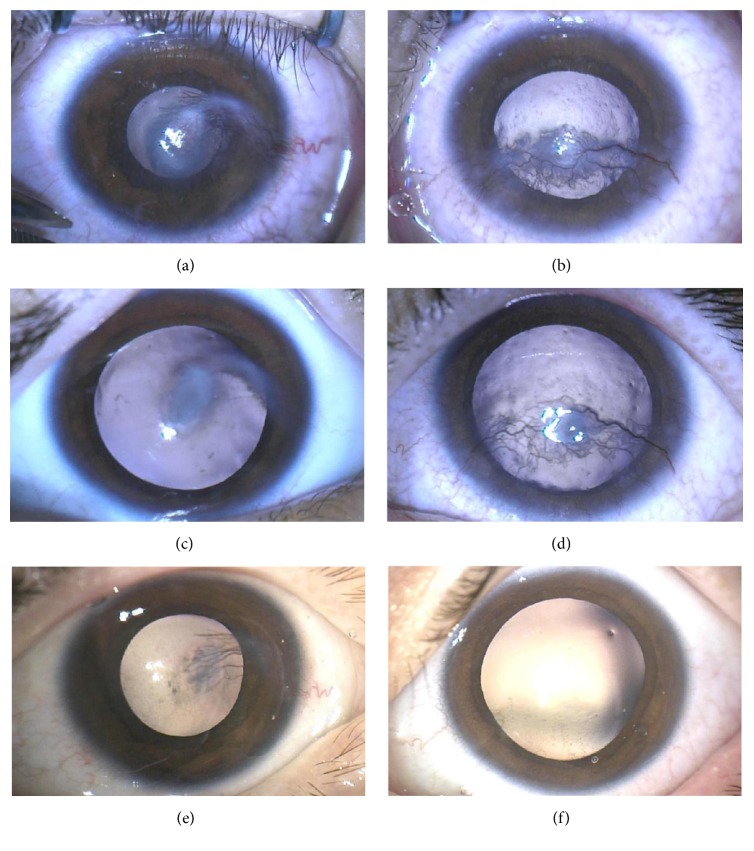
Photographs of Twin 2 with blepharokeratoconjunctivitis before and after treatment over an 8-month period. (a) Right cornea with stromal and epithelial vessels. (b) Left cornea with stromal and epithelial vessels. (c) Right cornea four weeks after subconjunctival triamcinolone and bevacizumab injections showing regressing vessels. (d) Left cornea in four-week time with no injections showing persisting stromal and epithelial vessels and no observable benefit from systemic effect of bevacizumab. (e) Right cornea 8 months after subconjunctival triamcinolone and bevacizumab injections and 5 months after additional triamcinolone injections, with corneal scarring. (f) Left cornea 7 months after subconjunctival triamcinolone and bevacizumab injections and 5 months after additional triamcinolone injections, with corneal scarring.

Twin 2 had a history of right esotropia and was undergoing part-time occlusion therapy (right eye: +3.00, left eye: −0.50/+2.50 × 90). He presented with frequent blinking of the right eye. BCVA was 20/400 in the right and 20/50 in the left. EUA showed bilateral stromal corneal neovascularization, without epithelial defect (Figures 2(a)-2(b)). There were blepharitis and conjunctivitis bilaterally. Like his brother, Twin 2 was investigated for other causes of interstitial keratitis and corneal neovascularization (Appendix 1).

Twin 2 underwent the same treatment as his brother including conventional treatment and subconjunctival triamcinolone and bevacizumab injections resulting in corneal scarring and suppressed blood vessels bilaterally (Figures 2(c)–2(f)). BCVA 10 months after presentation was 20/40 in the right and 20/50 in the left eye. IOP was 9mmHg in the right and 10mmHg in the left eye.

## 3. Discussion

Although conventional therapies for BKC have well-demonstrated efficacy, nonadherence can lead to chronic debilitating symptoms due to corneal scarring [1, 2, 4]. The issue of compliance was factored into the decision for the twins to receive subconjunctival therapy, as they were developmentally delayed and undergoing amblyopia therapy. The need for multiple EUA carries anaesthetic-related risks and costs that need to be weighed against the cost of blindness.

Subconjunctival triamcinolone and bevacizumab have been used with success for many conditions with corneal neovascularization [5–7]. One case series has described sub-tenon triamcinolone use for BKC, which produced resolution of symptoms, diminished corneal haze, and improved BCVA [8]. In our case series, each patient's left eye served initially as an internal control, strengthening the causal link between the triamcinolone and bevacizumab injections and regression of corneal neovascularization. The management is unique as it involved simultaneous use of these agents in children. Combined medication usage also makes it difficult to determine whether the therapeutic effect occurred because of the bevacizumab alone or the triamcinolone alone or whether both were needed. However, to our knowledge, there has been no literature to date describing simultaneous use of subconjunctival bevacizumab and subconjunctival triamcinolone in patients with BKC.

The advantage of subconjunctival therapy is that it is long acting, improving compliance. There may be bilateral response after unilateral subconjunctival bevacizumab injection; however this was not observed in the two cases presented [9, 10]. The disadvantages of bevacizumab are the potential for persistent corneal epithelial defects or thinning and systemic adverse effects [7]. Triamcinolone may not be readily available and may cause cataract and glaucoma [5].

Both twins received subconjunctival triamcinolone and bevacizumab with improvement in symptoms and partial regression of corneal neovascularization.

## Supplementary Materials

Supplementary Materials Appendix 1. Investigations for the twins with blepharokeratoconjunctivitis. Full blood count (FBC), electrolytes urea and creatinine (EUC), calcium magnesium phosphate (CMP), liver function tests (LFT), C-reactive protein (CRP), immunoglobulin M (IgM), immunoglobulin G (IgG), herpes simplex virus (HSV), varicella-zoster virus (VZV), Epstein-Barr virus (EBV), cytomegalovirus (CMV),* Treponema pallidum* (TP) antibody test, tuberculosis (TB), anti-nuclear antibodies (ANA), anti-neutrophil cytoplasmic antibody (ANCA), erythrocyte sedimentation rate (ESR), white cell count (WCC), polymerase chain reaction (PCR), and Epstein-Barr encoding region in situ hybridization (EBER ISH).
