# Supplementary material for: Simultaneous Subconjunctival Triamcinolone and Bevacizumab Injections for Management of Blepharokeratoconjunctivitis in Children
Source: Case Rep Ophthalmol Med. 2018 Jun 5;2018:2602487. doi: 10.1155/2018/2602487 (PMC6008772; doi:10.1155/2018/2602487)
Supplement: Supplementary Materials — Appendix 1. Investigations for the twins with blepharokeratoconjunctivitis. Full blood count (FBC), electrolytes urea and creatinine (EUC), calcium magnesium phosphate (CMP), liver function tests (LFT), C-reactive protein (CRP), immunoglobulin M (IgM), immunoglobulin G (IgG), herpes simplex virus (HSV), varicella-zoster virus (VZV), Epstein-Barr virus (EBV), cytomegalovirus (CMV), Treponema pallidum (TP) antibody test, tuberculosis (TB), anti-nuclear antibodies (ANA), anti-neutrophil cytoplasmic antibody (ANCA), erythrocyte sedimentation rate (ESR), white cell count (WCC), polymerase chain reaction (PCR), and Epstein-Barr encoding region in situ hybridization (EBER ISH). [file 2602487.f1.docx]

**Appendix 1.** Investigations for the twins with blepharokeratoconjunctivitis.

| **Bloods** | **Twin 1** | **Twin 2** |
| --- | --- | --- |
| FBC, EUC, CMP, LFT, Coagulation profile, CRP* | normal | normal |
| HSV1 & HSV2 IgM & IgG* | negative | negative |
| VZV IgM* | negative | negative |
| VZV IgG* | negative | positive |
| EBV IgM* | negative | negative |
| EBV IgG* | negative | positive |
| CMV IgM & IgG | negative | negative |
| Lyme borrelia | negative | negative |
| Syphilis TP | negative | negative |
| TB (Gamma Interferon) | negative | negative |
| Lead, Zinc, Vitamin A | normal | normal |
| ANA, ANCA, Rheumatoid factor, ESR | negative | negative |
| **T cell subsets** |  |  |
| WCC | 8,350 x10^6/L (normal) | 10,630 x10^6/L (normal) |
| Lymphocyte count | 5,094 x10^6/L (high) | 6,038 x10^6/L (high) |
| **Conjunctival swabs** |  |  |
| HSV PCR | negative | negative |
| VZV PCR | negative | negative |
| EBV PCR | negative | negative |
| Acanthamoeba | negative | negative |
| EBER ISH | negative | negative |
| **Corneal swabs** |  |  |
| HSV PCR | negative | negative |
| VZV PCR | negative | negative |
| Chlamydia PCR | negative | negative |
| **Eyelid conjunctival biopsy histopathology** | non-granulomatous, non-specific acute-on-chronic inflammation | non-specific acute-on-chronic inflammation, with an isolated non-necrotising granuloma |

*Repeated

Full blood count (FBC), Electrolytes urea and creatinine (EUC), Calcium magnesium phosphate (CMP), Liver function tests (LFT), C-reactive protein (CRP), Immunoglobulin M (IgM), Immunoglobulin G (IgG), Herpes simplex virus (HSV), Varicella-Zoster virus (VZV), Epstein-Barr virus (EBV), Cytomegalovirus (CMV), Treponema Pallidum antibody test (TP), Tuberculosis (TB), Antinuclear antibodies (ANA), Antineutrophil cytoplasmic antibody (ANCA), Erythrocyte sedimentation rate (ESR), White cell count (WCC), Polymerase Chain Reaction (PCR), Epstein-Barr encoding region in situ hybridization (EBER ISH).
